# Comparative Lipidomic Profiling of Camel and Cow Milk from a Shared Semi-Desert Pasture: Implications for Camel Adaptation to Arid Environments

**DOI:** 10.3390/molecules31060952

**Published:** 2026-03-12

**Authors:** Lin Zhu, Xiushan Tan, Zhiwei Li, Qiuyue Gong, Gengyan Duan, Changjiang Zang, Yong Chen, Fengming Li

**Affiliations:** 1 College of Animal Science, Xinjiang Agricultural University, Urumqi 830052, China; 2 Culinary and Catering Management, Xinjiang Vocational University, Urumqi 830013, China

**Keywords:** camel milk, cow milk, lipidomics, oxidized lipids, differential lipid compounds

## Abstract

This study reveals lipidomic adaptations in camel milk that are crucial for neonatal development in desert environments. Using UHPLC-MS/MS and targeted oxylipidomics, we compared milk from free-grazing camels and cows from the same region. We identified 2460 lipids across 44 subclasses and 11 oxygenated lipids in three groups. Glycerophospholipids (GP) were dominant in both. We found 498 differentially expressed lipids, including potential biomarkers such as phosphatidylinositol (PI 18:0/22:3), phosphatidylethanolamine (PE 18:0/22:3), and two triacylglycerol (TG) species. Camel milk was dominated by phosphatidylcholine (PC, approximately 49%) and PI (approximately 22%), whereas cow milk was predominantly composed of TG (nearly 98%). Pathway analysis showed 11 key altered lipid pathways, mainly glycerophospholipid metabolism. These results define camel milk’s unique lipid profile—linked to desert adaptation—and provide molecular insights into its role in supporting neonatal camels in arid environments.

## 1. Introduction

Milk is the primary source of nutrition for mammalian neonates, and its composition has evolved to meet the specific ecological and developmental demands of each species [1]. In arid and semi-desert ecosystems, camel milk represents a unique nutritional matrix that supports calf growth under extreme temperature fluctuations, water scarcity, and limited forage availability. In extreme aridity, where other ruminants struggle to thrive, the resilience of camels is of particular ecological and agricultural importance. In contrast to the catastrophic declines observed in cattle, sheep, and goats during persistent droughts, camels are uniquely resilient, demonstrating an unsurpassed capacity for long-term milk production under such extremes [2,3].

Similar to other milk types, the composition of camel milk lipids is influenced by numerous environmental and physiological factors [4,5,6]. Season, genetics, health status, and husbandry conditions are also critically important [7,8]. Seasonal variations in pasture quality lead to differences in fatty acid composition. Under pure grazing conditions, where animals rely solely on natural pastures without supplemental concentrate, the composition of milk fat depends strongly on the plant species present in the sward [5]. As a pseudo-ruminant, the camel exhibits distinct digestive characteristics compared with cattle. The camel’s gastrointestinal architecture enables more efficient utilization of crude fiber, allowing it to extract greater amounts of fatty acid precursors from low-quality forages. Consequently, camel milk has a high proportion of long-chain fatty acids, approximately 96.4%, whereas in cow milk it is 85.3% [9]. Under identical grazing conditions, cattle, in contrast, display a higher synthesis rate of short-chain fatty acids, particularly butyrate and caproate, reflecting the mammary gland’s direct utilization of acetate and butyrate [10]. Milk lipid composition is one of the most plastic nutritional traits through which mammals adapt neonatal development to environmental constraints.

However, differences in fatty acid composition alone provide limited insight into the structural organization and functional potential of milk lipids. Lipid constituents are central to milk’s functional attributes. They serve as dense energy reservoirs, structural components of cell membranes [11], and precursors for intracellular signaling molecules [12]. Beyond their role as energy substrates, many milk lipids exert biological functions through their molecular structures and lipid-class-specific organization. Moreover, specific lipid species have been implicated in regulating intestinal homeostasis and neurodevelopment, processes critical for neonatal survival in harsh environments. Notably, these functions are primarily mediated by complex lipid species, potentially including glycerophospholipids and oxidized lipids. Oxidized lipids, oxygenated metabolites of polyunsaturated fatty acids, are key mediators of inflammation, the oxidative stress response, and immune regulation, processes that are highly relevant to survival in arid environments.

Comparative lipidomic investigations have revealed that camel milk differs markedly from cow milk, exhibiting smaller fat globules [13,14,15], a higher proportion of unsaturated fatty acids [16], and an enriched phospholipid fraction [17]. However, most previous studies have examined milk from distinct geographic or management systems, making it difficult to disentangle species-specific lipid signatures from environmental influences. These observations are primarily descriptive and do not elucidate differences in individual lipid species or metabolic pathways among species within the same region.

To address these limitations, we performed the first comprehensive comparative lipidomic and oxylipidomic analysis of camel and cow milk collected under identical grazing conditions. Using ultra-high-performance liquid chromatography–tandem mass spectrometry (UHPLC-MS/MS) combined with targeted oxylipidomics, we delineated differences in lipid subclasses and major categories, identified potential species-discriminatory biomarkers, and analyzed the associated enriched pathways. The findings underscore the biochemical adaptive role of camel milk in supporting neonatal survival under arid conditions and establish a robust comparative framework for future cross-species research in milk lipid biology.

## 2. Results and Discussion

### 2.1. Analytical Method Validation

Methodological robustness was confirmed through quality control (QC) samples pooled from equal aliquots of all camel and cow milk extracts. Total ion chromatograms (TICs) in both positive and negative ion modes showed consistent peak distributions, with retention time shifts of <0.05 min (Figure S1). Multivariate analysis revealed that QC samples clustered tightly within two standard deviations of the PCA score plot (Figure S2), positioned at an intermediate point along PC1 between the camel and cow milk groups. More than 80% of detected lipids showed RSD < 20% in QC samples (Figure S3). This confirms the analytical platform’s reproducibility and stability.

### 2.2. Comparative Lipidomic Profiling

**Figure 1:**
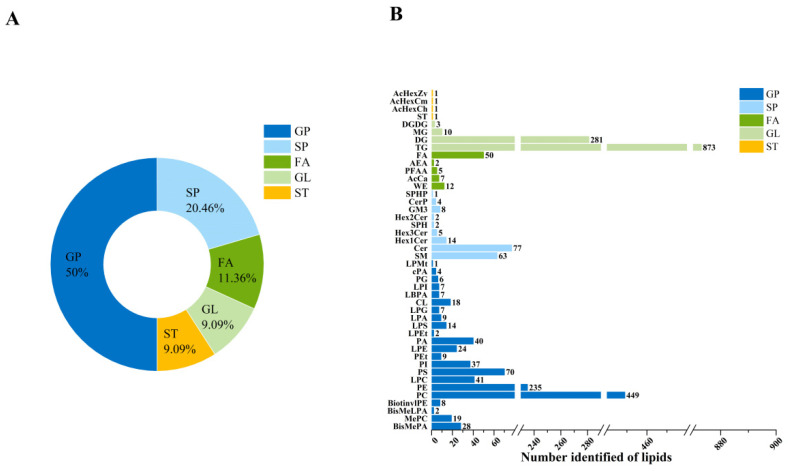
The number of identified species within each category relative to the total number of identified lipid species (**A**). Number of lipid species identified in 5 classes and 44 lipid subclasses (**B**).

Untargeted lipidomic analysis identified a total of 2460 lipid compounds in camel and cow milk, 1792 in positive ion mode and 668 in negative ion mode. Each “identified species” represents a distinct lipid molecular entity, confirmed by high-confidence matching in LipidSearch 5.1 software. Lipids were classified into five major classes based on the LIPID MAPS Consortium: glycerophospholipids (GPs, 50.00%), sphingolipids (SPs, 20.46%), fatty acids (FAs, 11.36%), sterol lipids (STs, 9.09%), and glycerolipids (GLs, 9.09%) (Figure 1A). GP constituted the predominant lipid class in milk, underscoring its central role in milk lipid composition. These phospholipids envelop the triacylglycerol (TG) core, thereby forming a unique and structurally complex lipid transport system [18,19,20]. According to Garcia et al. [17], the total phospholipid content in camel milk was quantified at 0.503 mM, showing significantly higher levels than those reported in human (0.324 mM), cow (0.265 mM), and equine milk (0.101 mM). As major components of cellular membranes, GPs also serve as important signaling molecules. Beyond influencing membrane properties, certain GPs bind to specific receptors and activate downstream biological pathways [21]. They are implicated in a range of physiological processes, including calcium homeostasis, protein translocation, apoptosis, inflammation, and neurogenesis [22].

Subclass analysis identified 44 lipid subclasses (Figure 1B), with TG (35.49%, 873 species), PC (18.25%, 449 species), and diacylglycerols (DG, 11.42%, 281 species) dominating. Phospholipid diversity was pronounced, with phosphatidylethanolamine (PE, 9.55%, 235 species), phosphatidylserines (PS, 2.85%, 70 species), and phosphatidylinositols (PI, 1.50%, 37 species) collectively accounting for 13.9% of the lipidome. SP were moderately represented, including ceramides (Cer, 3.13%, 77 species) and methyl phosphatidylcholines (MePC, 0.77%, 19 species). This detailed classification provides a comprehensive overview of lipid composition, underscoring the diversity and distribution of lipid species in the analyzed samples. TG, PC, DG, and PE emerged as the predominant lipid classes defining the overall milk lipid profile.

Notably, several lipid subclasses were detected at trace levels (<1%), including Hex1Cer (0.57%), Hex2Cer (0.08%), and PS (2.85%). Despite their low abundance, these lipids may exert important biological regulatory functions [23,24,25]. Of particular interest was the detection of sphingolipid metabolism-related species (SPH, SPHP) and anandamide (AEA), suggesting the potential involvement of specific signaling pathways in milk [26,27,28,29,30].

While MS/MS data enable the identification of lipid classes and their summed compositions, elucidating their complete structures (e.g., the sn-positional assignments of acyl chains in triglycerides) still requires specialized analytical approaches. Furthermore, among the 2460 lipids initially identified, many belong to relatively rare categories. Therefore, future studies should not only expand the sample size but also place particular emphasis on validating these lipids and their structural details, thereby accumulating sufficient data to further explore their biological significance.

### 2.3. Multivariate Discrimination of Milk Lipidomes

**Figure 2:**
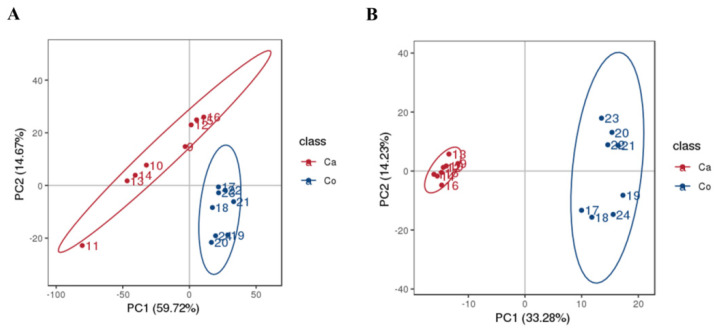
Plot of PCA scores of lipid compounds in camel and cow milk in positive (**A**) and negative (**B**) ion modes.

**Figure 3:**
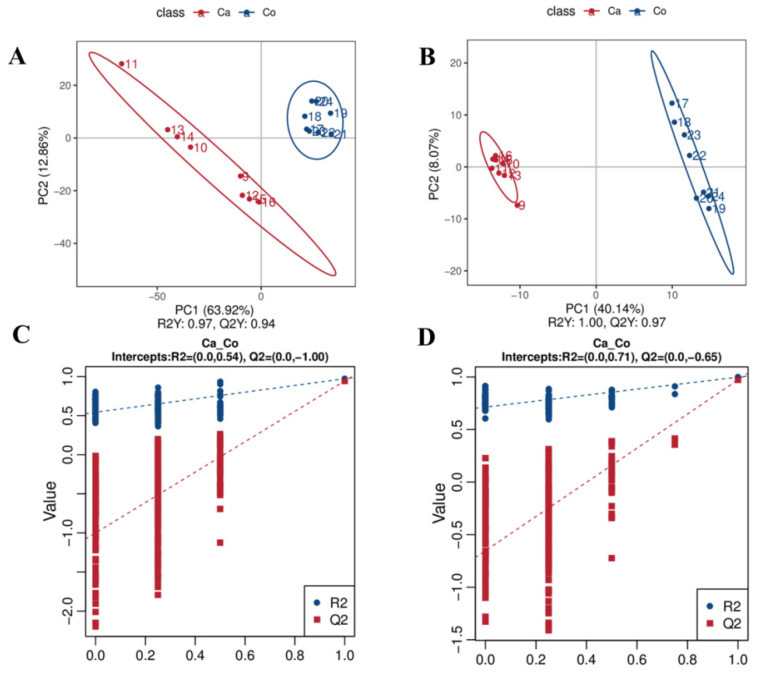
PLS-DA score plot and PLS-DA displacement test plot in positive (**A**,**C**) and negative (**B**,**D**) ion mode.

PCA and PLS-DA revealed significant lipidomic divergence between camel and cow milk. Clear group separation was observed in both positive (PC1: 59.72%, PC2: 14.67%) and negative (PC1: 33.28%, PC2: 14.23%) ion modes (Figure 2). The PLS-DA model exhibited high predictive accuracy (Q^2^ > 0.94) and reliability (permutation test, *p* < 0.05) (Figure 3), confirming the discriminative capacity of the lipidomic profiles and establishing a robust framework for species-specific biomarker identification.

### 2.4. Identification of Differential Lipid Species

**Figure 4:**
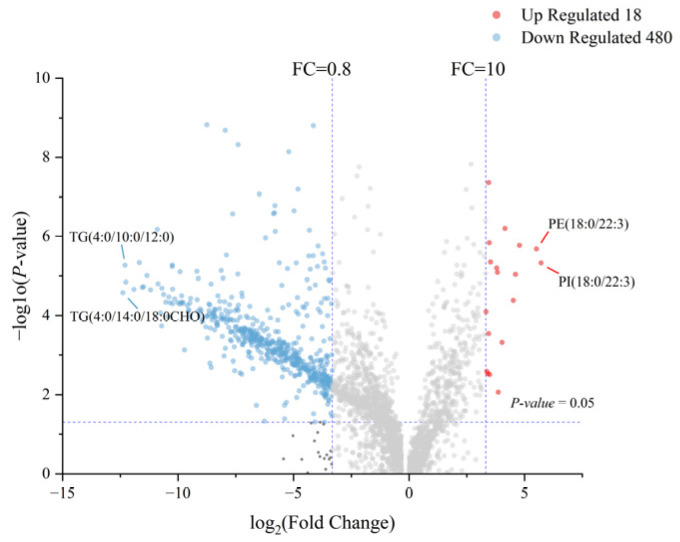
Volcano plot of 498 differential lipids. Red indicates upregulation in camel milk relative to cow milk, while blue indicates downregulation, gray indicates no significant difference, and the purple dashed lines represent the thresholds for fold change and statistical significance.

**Figure 5:**
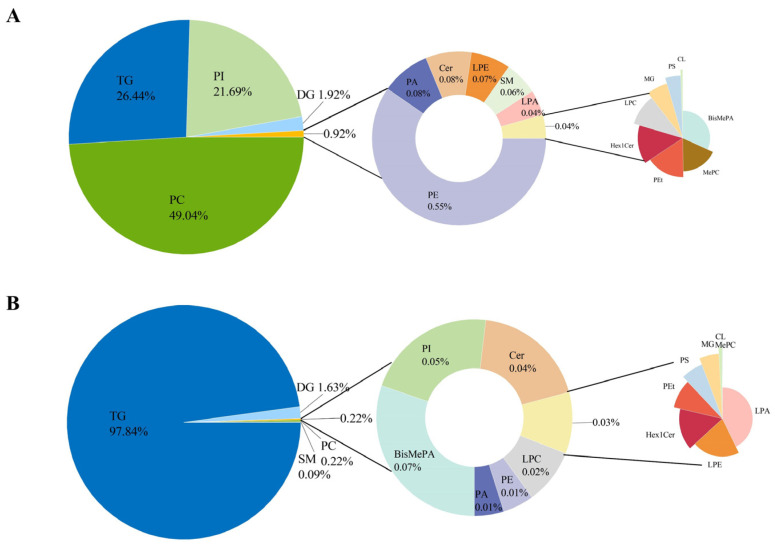
Abundance of lipid subclasses in camel milk (**A**) and cow milk (**B**).

Using a multi-criteria statistical framework (*p* < 0.05, FC > 10, and VIP > 1.0), 498 differential lipid species spanning 18 subclasses (Table S1)—including TG, DG, PC, PE, Cer, and sphingomyelin (SM)—were identified. Camel milk showed the significantly higher abundance (*p* < 0.05) of 18 lipid species, all belonging to the glycerophospholipid class. These comprised six PCs—PC (29:1), PC (16:1/18:3), PC (14:1/20:3), PC (34:4), PC (14:1/18:3), and PC (18:3/16:1); six PEs—PE (18:0/22:3), PE (18:1/22:3), PE (17:1 CHO/20:4), PE (18:2/22:3), PE (16:1/20:4), and PE (18:2/22:2); two PIs—PI (18:0/22:3) and PI (18:0/20:3); one lysophosphatidic acid—LPA (16:0); one lysophosphatidylethanolamine—LPE (20:3); and one phosphatidic acid—PA (16:0/18:0). In contrast, cow milk exhibited significantly higher abundance (*p* < 0.05) of 480 lipid species (Figure 4 and Table S2); among these, 411 were TGs. We quantified the abundance of milk lipid subclasses using normalized peak areas. In camel milk, PC (49.04%) and PI (21.69%) dominate, whereas in cow milk, PC, PE, and SM each comprise <1% of total lipids, with TGs accounting for 97.84% (Figure 5). Thus, camel milk is enriched in glycerophospholipids—particularly PC and PI, key components of the milk fat globule membrane—contrasting sharply with the TG-driven profile of cow milk. The markedly lower TG proportion and elevated phospholipid content in camel milk underscore its compositional uniqueness, suggesting that heightened energy storage demands in milk lipid metabolism suppress phospholipid biosynthesis and highlighting the complementary regulatory roles of these lipid classes [31].

These findings corroborate earlier reports of phospholipid enrichment in camel milk [17,32] and offer a more detailed, quantitative perspective. TGs, which serve as efficient energy-storage molecules, provide calves with a rapid energy source that aligns with the high-energy demands of temperate husbandry systems [33,34]. Camel milk exhibits a phospholipid-rich composition, suggesting a distinct lipid-based strategy suited to neonatal camel development under desert conditions. PC has been shown to modulate insulin receptor phosphorylation by altering the lipid organization of adipocyte plasma membranes, thereby influencing insulin sensitivity and glucose homeostasis [35]. In camels, this mechanism may promote energy efficiency, fat storage, and mitigation of metabolic stress, while providing a physiological defense against pathogens that impair development. Phospholipid-derived mediators, including PI, participate in cellular signaling pathways and may help neonatal camels cope with metabolic stressors such as heat and dehydration in extreme environments [36,37,38,39,40]. The amphiphilic nature of phospholipids may also enhance lipid absorption efficiency [17,32]. TG and DG are commonly found in milk from ruminants such as cows, buffaloes, goats, and yaks [41]. Their consistent presence suggests that TG and DG may serve as potential biomarkers for identifying traditional ruminant milk sources [41]. Overall, lipid subclass profiles vary markedly by milk origin, reflecting species-specific regulation of lipid metabolism.

Camels inhabiting arid regions primarily consume xerophytic and halophytic vegetation, such as species from Poaceae, Populus, and Amaranthaceae [42]. These dietary adaptations are likely responsible for the distinct lipid composition of camel milk, which in turn supports the survival and development of neonatal camels in challenging desert ecosystems.

### 2.5. Candidate Biomarker Discovery and Validation

**Figure 6:**
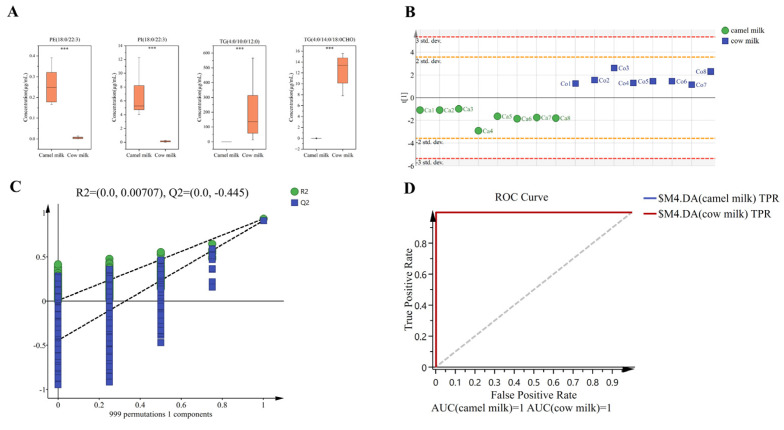
Comparison of the levels of four potential biomarkers levels between camel and cow milk (**A**), *** means *p* < 0.001; plot of OPLS-DA (using one axis) scores for potential biomarkers (**B**) and the corresponding 999 iterations of permutation tests (**C**) and receiver operator characteristic (ROC) curves (**D**).

To identify discriminative lipid candidate biomarkers, we ranked lipid compounds by FC values between the two milk types. Camel milk exhibited extreme enrichment of PI (18:0/22:3) (FC = 52.469) and PE (18:0/22:3) (FC = 45.667) while cow milk showed dominant upregulation of TG (4:0/14:0/18:0 CHO) (FC = 5383.916) and TG (4:0/10:0/12:0) (FC = 5040.083) (Figure 6A). The exceptionally high fold change values for these TGs in cow milk are primarily attributed to their near-zero abundance in camel milk, which results in a denominator near the detection limit during FC calculation. Our data preprocessing included appropriate handling of low-abundance signals to ensure robust statistical analysis. These pronounced differences support their utility as candidate biomarkers for the effective differentiation of camel and cow milk, providing a valuable basis for developing accurate authentication methods.

Using quantitative concentration data for these candidate biomarkers, OPLS-DA was employed to construct classification models for the four lipid species, achieving clear separation between camel and cow milk samples along the t [1] axis (Figure 6B). To evaluate model robustness, a permutation test with 999 permutations was performed (Figure 6C). The permutation test results, with R^2^ intercept values of 0.00707 and Q^2^ intercept values of −0.445, indicate that the OPLS-DA models are valid and not overfitted, demonstrating robust predictive capability. Furthermore, receiver operating characteristic (ROC) analysis of the four candidate lipid candidate biomarkers showed outstanding classification performance, with the combined biomarker panel achieving an area under the curve (AUC) of 1.0 (Figure 6D). The outstanding classification performance confirms the utility of lipid biomarkers in distinguishing camel and cow milk lipidomes. Similarly, lipidomic analyses have identified lactation-stage-specific biomarkers—such as two PEs and two Hex2Cers that differentiate bovine colostrum from mature milk [43], and two TGs, one DG, and one FFA that distinguish human from ewe colostrum [44]. Our findings establish four phospholipid/triacylglycerol biomarkers that reliably differentiate camel from cow milk under the tested conditions (Figure 6). These biomarker profiles are consistent with the phospholipid-enriched nature of camel milk and the triacylglycerol-dominated composition of cow milk. It should be noted that the sample size in this study (*n* = 8 per group) is relatively small. Although the OPLS-DA model showed excellent separation, its performance must be interpreted with caution because of the risk of overfitting. Future studies with larger and more diverse cohorts are needed to validate these candidate discriminative lipids as reliable markers.

### 2.6. Comparative Oxidized Lipidomics Analysis

To elucidate lipid alterations in camel milk associated with environmental factors that may reflect adaptation, we performed targeted quantitative analysis of oxidized lipids based on the results of untargeted lipidomics. Applying the criteria of FC > 1.5 or < 0.667 with *p* < 0.05, we identified three categories of oxidized lipids derived from PUFAs, comprising six arachidonic acids (AAs), three EPAs, and three DHA species (Table 1). Camel milk contained significantly higher levels of PUFA derivatives, notably 11,12-diHETrE (FC = 4.5) and 5-HEPE (FC = 2.4), whereas cow milk exhibited elevated oxidized lipids such as 8-HETrE (FC = 0.35).

Arachidonic acid-derived diols, including diHETrE, generally display low biological activity; however, several studies have reported their unique functions [45]. For instance, 11,12-diHETrE suppresses NF-κB pathway activation and reduces the release of inflammatory mediators [46]. Meanwhile, 5-HEPE, an EPA metabolite, activates GPR120 receptors, thereby promoting IL-10 production while inhibiting NLRP3 inflammasome activation [47]. These molecular mechanisms align with the critical demands of mitigating oxidative stress and maintaining immune homeostasis in camels inhabiting extreme environments, suggesting that the camel milk lipidome represents an adaptive response to environmental stressors. Consequently, the presence of polyunsaturated fatty acids (PUFAs) and their oxidized metabolites —such as EPA-derived oxylipins—in camel milk may contribute to enhanced oxidative stress tolerance in desert settings [48,49].

The compositional differences in milk may be attributed to extensive biohydrogenation during ruminal digestion in cows, which reduces the abundance of unsaturated fatty acids in milk [50]. In contrast, as pseudo-ruminants, camels retain higher proportions of unmodified unsaturated fatty acids, thereby preserving their bioactive oxidized derivatives. This digestive metabolic divergence likely underlies the distinct oxylipid profiles observed between camel and cow milk [50].

### 2.7. Lipid Correlation Networks

**Figure 7:**
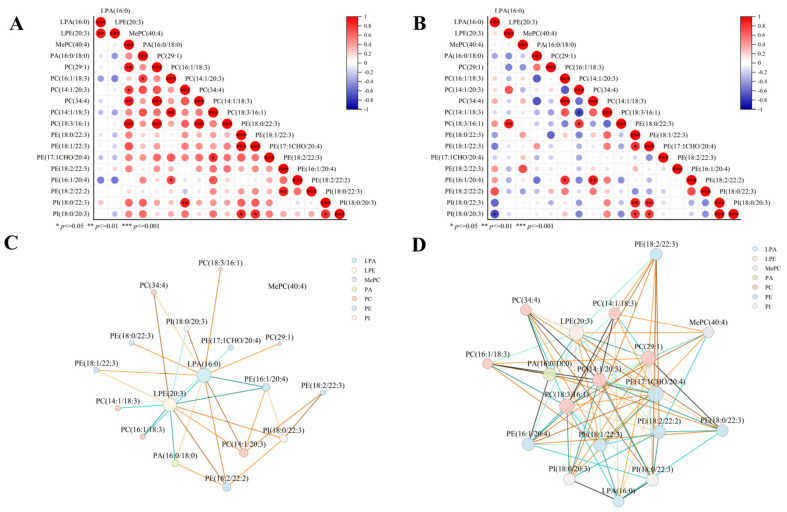
Analysis of differential lipid correlation and chord between camel (**A**,**C**) and cow milk (**B**,**D**).

Comprehensive lipidomic analysis revealed strong within-species correlations among lipids, with particularly pronounced interactions within lipid subclasses, suggesting potential metabolic associations. To systematically evaluate these lipid interactions, we performed Pearson correlation analysis and visualized relationships among 18 key lipids that are significantly upregulated in camel milk in a heatmap, comparing their profiles between camel and cow milk (Figure 7). Weighted correlation network analysis revealed a network of 171 correlations, with camel milk containing 36 positive correlations versus 31 in cow milk (*p* < 0.05; Figure 7C,D). Our results demonstrated strong subclass-specific interactions, particularly within PC, PE, and PI classes. Given the established roles of PE and PC in cholesterol absorption and intestinal maturation, these findings underscore the functional significance of milk lipid networks and their broader nutritional implications [51,52,53]. Specifically, the tightly coordinated phospholipid modules in camel milk may potentially contribute to neurodevelopment and gut health adaptation in arid environments, warranting further functional investigations.

### 2.8. KEGG Pathway Enrichment Analysis

**Figure 8:**
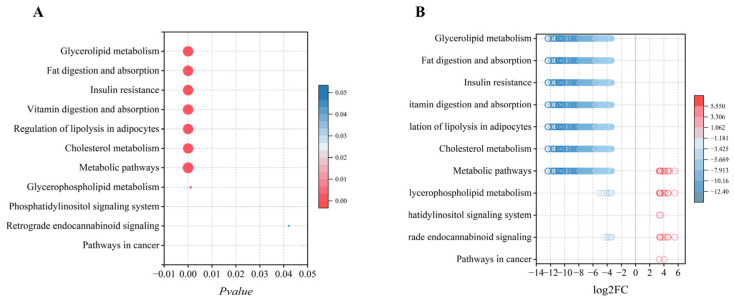
KEGG pathway analysis (**A**) and lollipop plot (**B**) of differential lipid compounds between camel and cow milk; Red: upregulated in camel milk, Blue: downregulated in camel milk.

To elucidate the potential relationship between differentially expressed lipids and metabolic pathways in camel milk, we conducted pathway enrichment analysis on identified lipid species. Given that KEGG pathways are primarily gene-centric, our analysis associates lipid composition with pathways annotated in KEGG, providing insights into potential metabolic alterations. Following annotation with relevant databases, pathway and enrichment analyses were performed based on topology-based pathway impact factors and *p*-values. The top 20 pathways ranked by *p*-value were selected and visualized. Comparative analysis of camel versus cow milk identified 35 KEGG metabolic pathways (Table S3), of which 11 were significantly enriched lipid metabolism pathways (Figure 8), reflecting functional divergence between the camel and cow milk lipidomes. Among these, seven pathways—including fat digestion and absorption—exhibited the most prominent clustering, indicating their central role in lipid metabolism. The adaptive regulation of GP, ST, and GL metabolism appears to converge on key pathways, including glycerophospholipid, glycerol, and cholesterol metabolism [11].

Camel milk lipids showed significant enrichment in glycerophospholipid metabolism (*p* = 0.001) and the phosphatidylinositol signaling system (*p* = 0.029). This enrichment aligns structurally with the high abundance of PC (49.04% of differential identified lipids) and PE (21.69% of differential identified lipids), both of which are critical for membrane biogenesis and synaptic plasticity [54,55]. Notably, all 18 differentially abundant lipid species identified in camel milk belonged to the GP class, which led to the observed enrichment of GP-centric metabolic pathways. This finding is consistent with compositional differences and highlights the central role of glycerophospholipid metabolism in camel milk. In contrast, cow milk lipids were primarily enriched in energy-related pathways, such as fat digestion and absorption, glycerolipid metabolism, and insulin resistance, consistent with its TG-dominated composition (97.84%), which supports rapid energy mobilization for postnatal development in temperate environments [56,57,58].

## 3. Materials and Methods

### 3.1. Sample Collection

The milk collection was approved by the Animal Welfare and Ethics Committee of Xinjiang Agricultural University (Approval No. 2024018).

Milk samples were collected from eight healthy Junggar Bactrian camels (camel milk group, 7–8 years old, 450.43 ± 56.23 kg) and eight Xinjiang brown cattle (cow milk group, 2–4 years old, 475.48 ± 37.3 kg), in Jiang’abulake Village, Yumin County (E 82.884197, N 46.170966), Xinjiang, China. Camels and cows were clinically healthy, and the criteria adopted for dam selection were similarity in body weight, age, and breed order. All animals were lactating females sampled at 2–3 months postpartum. Under free-grazing conditions on semi-desert steppe (an ecotone between desert and typical steppe), diets comprised drought-tolerant vegetation dominated by *Artemisia* spp. (*sagebrush*) and *Stipa* spp. (*needlegrass*).

All milk samples were collected during the morning milking session (between 07:00 and 09:00) to minimize diurnal variation. Both camel and cow herds were sampled concurrently within this window to ensure temporal comparability. Full udder milking was performed for each animal. Milk was manually extracted, filtered through sterile gauze into 50 mL cryovials, flash-frozen in liquid nitrogen, and stored at −80 °C until analysis. Each sample was processed individually without pooling to ensure biological replicates.

### 3.2. Instrumentation and Reagents

Chromatographic Systems: UHPLC: Vanquish™ (Thermo Fisher Scientific, Hennigsdorf, Brandenburg, Germany); LC: ExionLC™ AD (SCIEX, Framingham, MA, USA); columns: Accucore C30 (150 × 2.1 mm, 2.6 μm; Thermo Fisher Scientific, Hennigsdorf, Brandenburg, Germany); Atlantis Premier BEH C18 (10 cm × 2.1 mm; Waters, Milford, MA, USA); mass spectrometers: Q Exactive™ HF-X (Orbitrap™; Thermo Fisher Scientific, Hennigsdorf, Brandenburg, Germany); QTRAP^®^ 6500+ (SCIEX, Framingham, MA, USA); auxiliary equipment: ST16R Centrifuge (Thermo Fisher Scientific, Hennigsdorf, Brandenburg, Germany); Reacti-Therm Nitrogen Evaporator (Thermo Fisher Scientific, Hennigsdorf, Brandenburg, Germany); reagents: HPLC-grade solvents: acetonitrile, methanol, isopropanol, MTBE (Thermo Fisher Scientific, Hennigsdorf, Brandenburg, Germany); additives: formic acid, acetic acid, ammonium acetate; water: Ultrapure (18.2 MΩ·cm; Milli-Q, Millipore, Burlington, MA, USA); standards: SPLASH™ LIPIDOMIX™ (Avanti Polar Lipids, Birmingham, AL, USA), eicosanoids (Novogene, Beijing, China).

### 3.3. Sample Preparation

Lipid extraction from milk was adapted from the method of Matyash et al. [59] with modifications, mainly concerning sample volume and internal standard usage, specifically the use of a SPLASH™ LIPIDOMIX™ (Avanti Polar Lipids, Birmingham, AL, USA) internal standard mix and adjustment of solvent volumes for a 100 µL milk aliquot.

#### 3.3.1. Lipid Extraction

Aliquots (100 μL) of milk were homogenized with methanol (0.75 mL), MTBE (2.5 mL), and SPLASH™ internal standard (10 μL). After shaking (1 h, 25 °C), phase separation was induced with water (0.625 mL). The upper organic layer was collected after centrifugation (1000× *g*, 10 min). The lower phase was re-extracted with 1 mL of the solvent mixture (MTBE/methanol/water, 10:3:2.5 *v*/*v*/*v*), and the upper phase was collected. The combined organic phases were dried under N_2_ and reconstituted in isopropanol (100 μL) for LC-MS/MS analysis.

#### 3.3.2. Oxidized Lipid Extraction

Take a 100 μL milk aliquot, add 700 μL of 50 mM PBS, and centrifuge to collect the supernatant for standby. Add 700 μL of 10% methanol to the sediment and centrifuge to collect the supernatant for standby. Use a SPE (Atlantis Premier BEH C18, 10 cm × 2.1 mm; 1.7 µm particle size, Waters, Milford, MA, USA) desalination column, activate with 700 μL of methanol, centrifuge, and discard the filtrate. Add 700 μL of pure water. Centrifuge and discard the filtrate. Load the first 3 portions of supernatant in turn, centrifuge, and load each supernatant twice before discarding the filtrate. Add 700 μL of 10% methanol to the sample sediment, load it into the SPE desalination column, centrifuge, and discard the filtrate. All steps during oxidized lipid extraction were performed on ice to minimize lipid degradation and oxidation. Add 700 μL of methanol to elute the metabolite, collect the filtrate from this part, and freeze-dry.

### 3.4. UHPLC-MS/MS Analysis

To ensure data quality, procedural blanks (extraction without a sample) were included in each batch to monitor background contamination. All samples were analyzed in a single randomized injection sequence to minimize instrument drift bias. The stability of the analytical platform was monitored using QC samples interspersed throughout the run, with one QC sample injected every 3 experimental samples to ensure continuous monitoring and correction for instrument drift. The first two QC injections are used to monitor instrument status before sample injection and to equilibrate the chromatography–mass spectrometry system. The subsequent three QC injections are used for segmented scanning, and the resulting MS2 spectra, along with those from experimental samples, are used for lipid compound identification. The QC inserted in the middle of sample analysis is used to evaluate system stability throughout the experiment and to perform data quality control analysis.

#### 3.4.1. Untargeted Lipidomics

Ultra-high-performance liquid chromatography–tandem mass spectrometry (UHPLC-MS/MS, Thermo Fisher, Hennigsdorf, Brandenburg, Germany) analysis was carried out on a Vanquish UHPLC (Thermo Fisher, Hennigsdorf, Brandenburg, Germany) system [60] coupled to a Q Exactive™ HF-X (Orbitrap™; Thermo Fisher, Hennigsdorf, Brandenburg, Germany) mass spectrometer, using an Accucore C30 column (150 mm × 2.1 mm, 2.6 µm particle size; Thermo Fisher Scientific, Hennigsdorf, Brandenburg, Germany) maintained at 40 °C. The solvent gradient was programmed as follows: 30% B from 0 to 2 min, 43% B at 5 min, 55% B at 5.1 min, 70% B at 11 min, ramped to 99% B from 16 to 18 min, and then returned to 30% B at 18.1 min. The mobile phases consisted of A: acetonitrile/water (6:4) with 10 mM ammonium acetate and 0.1% formic acid and B: acetonitrile/isopropanol (1:9) with 10 mM ammonium acetate and 0.1% formic acid. Key MS parameters included polarity in both positive and negative modes, a spray voltage of ±3.5 kV, a capillary temperature of 320 °C, a scan range of *m*/*z* 114–1700, and HCD collision energies set at 22/24/28 eV.

#### 3.4.2. Targeted Oxidized Lipidomics

We used an ExionLC™ AD coupled to QTRAP^®^ 6500+ (SCIEX, Framingham, MA, USA), as well as a column equipped with an Atlantis Premier BEH C18 (10 cm × 2.1 mm; Waters, Milford, MA, USA). The gradient profile was as follows: 35% B at 0–0.5 min, which then linearly increased to 95% B at 9.5–10.5 min and 35% B at 11–14 min. The MS was operated in positive polarity mode with an IonSpray™ voltage (SCIEX, Framingham, MA, USA) of −4500 V, a temperature of 500 °C, and a curtain gas pressure of 0 psi.

### 3.5. Statistical Analysis

Data analysis was conducted using LipidSearch 5.1 software for peak alignment and lipid quantification, integrating precise mass, fragmentation patterns, and retention time. Redundancies, including adduct forms and isotopic variants, were removed during data processing to ensure each chemical entity was counted only once. Multivariate statistical analyses, including principal component analysis (PCA) and partial least squares-discriminant analysis (PLS-DA), were performed using the metaX platform. For PCA, we first applied a log2 transformation to the relative quantitative values in the meta intensity table and then used the PCA() function to perform centering and UV scaling (Z-score normalization). For PLS-DA, we similarly applied a log2 transformation to the relative quantitative values in the meta intensity table, followed by centering and further Z-score normalization (using the ’auto’ parameter). 

To detect differential lipids in the untargeted dataset, we applied False Discovery Rate (FDR) correction using the Benjamini–Hochberg method. Metabolites exhibiting significant alterations were identified based on a variable importance in projection (VIP) > 1.0, a *p*-value < 0.05, and an absolute fold change (FC) of at least 1. Results were visualized using heatmaps generated with the Pheatmap package in R 4.5.2, with z-score normalization applied to emphasize metabolite abundance patterns. Furthermore, correlation networks based on Pearson’s correlation coefficient were constructed and visualized via the corrplot R 4.5.2 package. For detailed lipid annotation, three major databases were used: the Kyoto Encyclopedia of Genes and Genomes (KEGG, https://www.genome.jp/kegg/pathway.html, accessed on 2 January 2026), the Human Metabolome Database (HMDB, https://hmdb.ca/metabolites, accessed on 2 January 2026), and LIPID MAPS (http://www.lipidmaps.org/, accessed on 2 January 2026).

## 4. Conclusions

In this study, we employed UHPLC-MS/MS-based untargeted lipidomics and a targeted oxylipidomics approach to characterize the lipid profiles of camel and cow milk. Our analysis identified 2460 lipid species, revealing profound compositional distinctions: 18 GPs were significantly enriched in camel milk, whereas 480 lipids dominated cow milk. Quantitative subclass profiling demonstrated that PC (49.04%) and PI (21.69%) constituted the major lipid classes in camel milk—contrasting sharply with the TG (97.84%)-predominant profile of cow milk, where PC, PE, and SM each represented < 1% of total lipids. Notably, four lipids—PI (18:0/22:3), PE (18:0/22:3), TG (4:0/14:0/18:0 CHO), and TG (4:0/10:0/12:0)—were identified as candidate biomarkers for discriminating between camel and cow milk.

Pathway enrichment analysis identified 11 significantly altered lipid metabolic pathways, largely linked to glycerophospholipid metabolism. These results may offer insights into the mechanistic basis of potential ecological adaptations of camel milk under arid conditions. Moreover, our comparative analysis suggests that camel milk-specific lipids are associated with metabolic pathways potentially relevant to desert adaptation. Collectively, these findings deepen our understanding of camel physiology in extreme environments and may suggest molecular mechanisms that could potentially contribute to their resistance against growth-impairing pathogens. The data generated in this study may serve as a useful reference for exploring potential strategies to enhance camel milk production and for future investigations into its possible nutritional and functional benefits.

## Figures and Tables

**Table 1:** Differential metabolites of lipid oxidation between camel and cow milk.

No	Name	Class	Relative Abundance (Camel Milk)	Relative Abundance (Cow Milk)	FC	*p*-Value	Up/Down
1	11,12-diHETrE	AA	134,878.8	29,850.0	4.5	<0.001	up
2	8-HDoHE	DHA	5060.3	1932.1	2.6	<0.001	up
3	DHA	DHA	2803.5	8050.4	2.5	<0.001	up
4	5-HEPE	EPA	3285.9	1900.6	2.4	<0.001	up
5	15-HEPE	EPA	5322.9	2263.5	1.7	<0.001	up
6	18-HETE	AA	320,987.5	128,003.8	1.7	<0.001	up
7	18-HEPE	EPA	1603.1	3245.5	1.7	<0.001	up
8	5-HETE	AA	14,840.4	23,793.8	0.6	<0.001	down
9	5,6-diHETE	AA	4485.5	2647.9	0.6	<0.001	down
10	PGF3a	AA	4142.3	2417.6	0.5	<0.001	down
11	8-HETrE	AA	552.3	891.2	0.3	<0.001	down

## Data Availability

The raw data generated in this study have been deposited in the public repository Mendeley Data and are accessible via the following link: https://data.mendeley.com/datasets/jj9s37gtzc/3 (accessed on 20 January 2026).

## Supplementary Materials

The following supporting information can be downloaded at https://www.mdpi.com/article/10.3390/molecules31060952/s1, Figure S1: Total ion chromatograms (TICs) acquired of camel milk and cow milk. Positive ion mode (A). Negative ion mode (B); Figure S2: Reliability of the analytical methods. (A,C) Plots of PCA and PCA3D scores for camel milk versus camel milk in positive ion mode. (B,D) Plot of PCA and PCA3D scores for camel’s milk versus cow’s milk in negative ion mode; Figure S3: The RSD values of the peak intensities in QC samples. Table S1: Lipid compounds with higher proportions in Ca group emulsions in positive and negative ion modes; Table S2: Lipid modes compounds with higher proportions in Co group emulsions in positive and negative ion modes; Table S3: Metabolic pathways identified in the lipids that were significantly different between camel milk and cow milk. Table S4: Lipids contents in camel milk and cow milk (ug/mL).

## Abbreviations

The following abbreviations are used in this manuscript:

AA	Arachidonic Acid
AEA	Anandamide
CL	Cardiolipin
Cer	Ceramide
DG	Diacylglycerol
DHA	Docosahexaenoic Acid
EPA	Eicosapentaenoic Acid
FA	Fatty Acid
GP	Glycerophospholipids
GL	Glycerides
Hex1Cer	Monohexosylceramide
Hex2Cer	Dihexyl yl formamide
LPC	Lysophosphatidylcholine
LA	Linoleic acid
LPE	Lysophosphatidylethanolamine
LPA	Lysophosphatidic acid
LC-PUFA	Long-chain polyunsaturated fatty acids
MePC	Methyl Phosphatidylcholine
MFGM	Milk fat globule membrane
PUFA	Polyunsaturated fatty acid
PS	Phosphatidylserine
PI	Phosphatidylinositol
PC	Phosphatidylcholine
PE	Phosphatidyleethanolamine
PA	Phosphatidyl acid
SP	Sphingolipids
SPH	Sphingol
SM	Sphingomyelin
SPHP	Phosphatidylinositol-4, 5-Diphosphate
ST	Sterol Lipids
TG	Triacylglycerol
