# Supplementary material for: Comparative Lipidomic Profiling of Camel and Cow Milk from a Shared Semi-Desert Pasture: Implications for Camel Adaptation to Arid Environments
Source: Molecules. 2026 Mar 12;31(6):952. doi: 10.3390/molecules31060952 (PMC13029572; doi:10.3390/molecules31060952)
Supplement: Supplementary file 1 [file molecules-31-00952-s001.zip › molecules-4160196-Supplementary Materials.pdf]

Supplementary Materials

Comparative Lipidomic Profiling of Camel and Cow Milk from a Shared Semi-Desert Pasture: Implications for Camel Adaptation to Arid Environments

Lin Zhu [1], XiushanTan [2], Zhiwei Li [1], Qiuyue Gong [1], Gengyan Duan [1], Changjiang Zang [1], Yong Chen[1] and Fengming Li [1*]

1 College of Animal Science, Xinjiang Agricultural University, Urumqi, 830052, China; 320222603@xjau.edu.cn (L.Z.); 15099325614@163.com (Z.L.); 13438778792@163.com (Q.G.); 17809239008@163.com (G.D.); zcj780@126.com (C.Z.); xjauCy@163.com (Y.C.)
2 Culinary and Catering Management, Xinjiang Vocational University, Urumqi 830013, China; t2807659761@163.com
* Correspondence: lifming@xjau.edu.cn

A

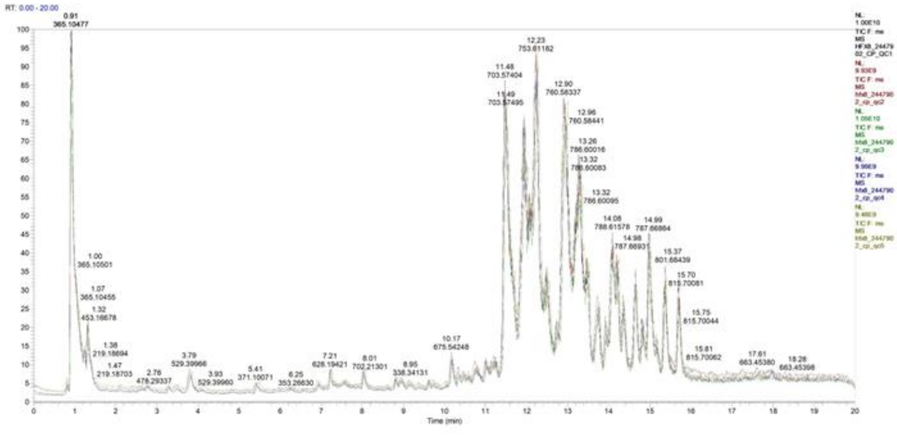

B

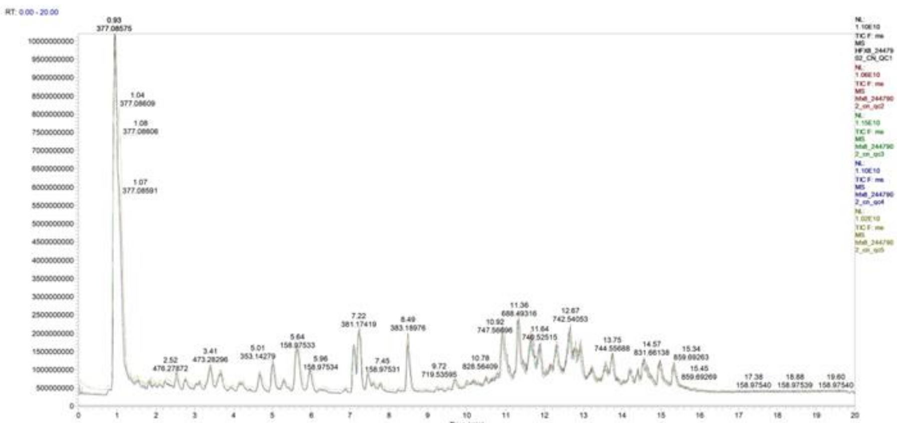

Figure S1. Total ion chromatograms (TICs) acquired of camel milk and cow milk. Positive ion mode (A). Negative ion mode (B).

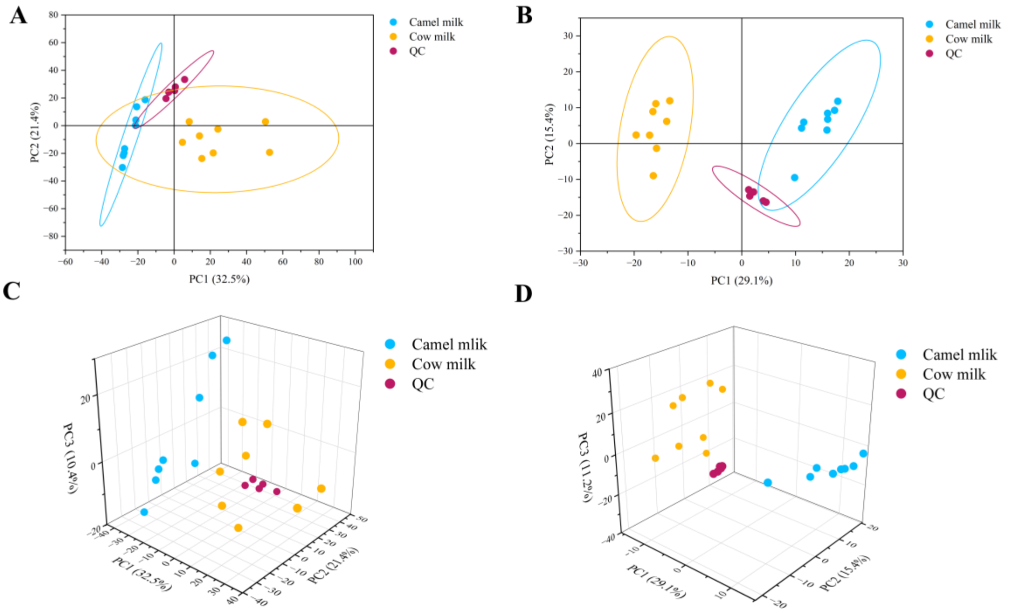

Figure S2. Reliability of the analytical methods. (A) (C) Plots of PCA and PCA3D scores for camel milk versus camel milk in positive ion mode. (B) (D) Plot of PCA and PCA3D scores for camel's milk versus cow's milk in negative ion mode.

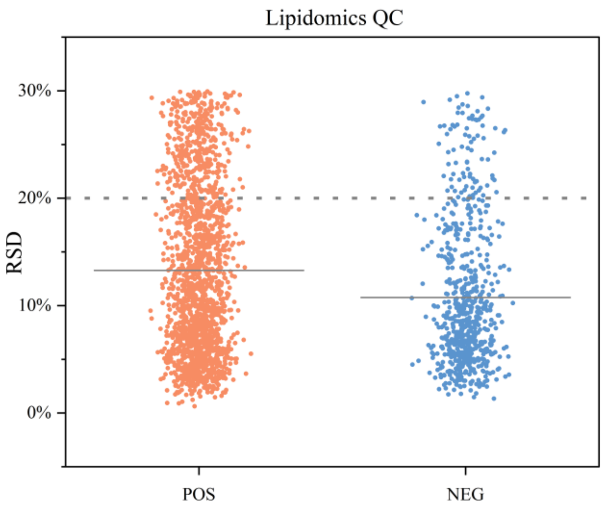

Figure S3. The RSD values of the peak intensities in QC samples.
